# Finding NEMO: The quest for next‐generation genetically encoded calcium indicators

**DOI:** 10.1002/ctm2.1428

**Published:** 2023-10-05

**Authors:** Wenjia Gu, Jia Li, Yuepeng Ke, Yubin Zhou, Youjun Wang

**Affiliations:** ^1^ Beijing Key Laboratory of Gene Resource and Molecular Development College of Life Sciences Beijing Normal University Beijing China; ^2^ Joint Laboratory of Opto‐Functional Theranostics in Medicine and Chemistry The First Hospital of Jilin University Changchun China; ^3^ Institute of Biosciences and Technology Texas A&M University Houston Texas USA; ^4^ Department of Translational Medical Sciences School of Medicine Texas A&M University Houston Texas USA; ^5^ Key Laboratory of Cell Proliferation and Regulation Biology Ministry of Education College of Life Sciences Beijing Normal University Beijing China

1

Calcium ion (Ca^2+^) serves as a ubiquitous second messenger within eukaryotic cells. Alterations in free Ca^2+^ concentration, known as Ca^2+^ signals,^1^ play a vital role in regulating a wide array of physiological and pathological processes, including synaptic transmission, muscle contraction, cytokine secretion, gene transcription, cell growth, and cell death.^2^ Abnormal Ca^2+^ signalling has been implicated in various human disorders such as cancer, immunodeficiency, myocardial hypertrophy, and neurodegenerative diseases.^3^ Ca^2+^ signals possess the capacity to convey crucial information through three‐dimensional variations in amplitude, temporal and spatial distribution (Figure 1A). Over the past four decades, considerable efforts have been devoted to developing biosensors and actuators aimed at interrogating these three fundamental properties of Ca^2+^ signals.^4^ Given their facile genetic manipulation and tunable sensitivity, genetically encoded Ca^2+^ indicators (GECIs) have gained widespread popularity for tracking intracellular Ca^2+^ signals or Ca^2+^‐modulated cell activities.^5^ A typical GECI comprises a Ca^2+^‐sensing moiety (sensor) and a fluorescence reporting module (reporter). Upon binding or dissociation of Ca^2+^, the sensor often undergoes conformational changes that modify the spectral properties and/or brightness of the reporter,^6^ thereby enabling real‐time observation of Ca^2+^ signals by quantifying changes in fluorescence intensities (*F*) (Figure 1B).

**FIGURE 1:**
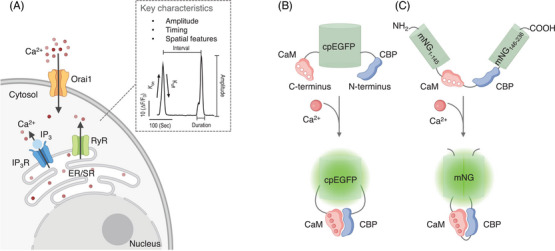
Basics of Ca^2+^ signals and design strategies of single fluorescence genetically encoded Ca^2+^ indicator (GECI). (A) Three key characteristics of Ca^2+^ signals, amplitude, timing and spatial properties. The timing features include on and off rate (K_on_, K_off_), duration of each transient, and intervals between Ca^2+^ pulses. Its spatial properties include the location and the spatial distribution of each signal. Some Ca^2+^ channels were shown as examples of sources of Ca^2+^ signals. Orai1 channel on the plasma membrane, the InsP_3_ receptors (IP_3_R) and ryanodine receptors (RyR) located on the endoplasmic reticulum (ER) membrane or sarcoplasmic reticulum(SR) membrane. The blue circle represents IP_3_. (B, C) Design strategies of GECIs: GCaMP, (B) and NEMO, (C). The red balls represent Ca^2+^ ions.

By driving their expression under tissue‐specific promoters or via fusion with the organelle‐targeting moieties, GECIs can be precisely expressed in particular cell types or subcellular compartments, hence facilitating the accurate reporting of spatial attributes of Ca^2+^ signal. Through several generations of evolution over the past two decades, GECIs have shown significantly enhanced sensitivity and diversified kinetics.^6^ Among them, the latest jGCaMP8f exhibits an impressive activation half‐time of 2 ms, which enables better decoding of fast‐acting Ca^2+^ signals seen in excitable cells including neurons and cardiomyocytes.^7^ Nonetheless, the advancements in their intracellular dynamics, particularly the capacity of GECIs to resolve subtle Ca^2+^ signal amplitudes, have progressed at a comparatively slower pace since the inception of the GCaMP6 series. The dynamics of GECIs are commonly gauged by the dynamic range (DR) or the signal‐to‐baseline ratio (SBR). DR represents the responsive capacity of GECI, which is calculated from its fluorescence intensity at Ca^2+^‐saturated (*F_max_*) or Ca^2+^‐free states (*F_min_*) and expressed as (*F_max_*—*F_min_*)/*F_min_*.^8^ By comparison, SBR is a more physiologically‐relevant metric, defined as the ratio of the change in the fluorescence intensity (*F—F*_0_, or *ΔF*) over the basal fluorescence intensity (*F*_0_). Among the green GECIs utilizing circularly permuted enhanced green fluorescent protein (cpEGFP) as the reporter (Figure 1B), the GCaMP6 series remains the most prevalent. The in‐cellulo DRs of GCaMP6 and its updated version jGCaMP7c measure in the range of 20–30,^9^ and the latest jGCaMP8s exhibits a DR of only 4.1. Overall, a notable technical gap persists in the field of GECIs, necessitating the creation of biosensors with increased DR or enhanced SBR. A technical leap in this direction will likely enable more accurate and sensitive detection of subtle fluctuations in Ca^2+^ signals.

The development of GCaMP variants with an expanded DR faces a significant hurdle due to the constraint imposed by the relatively low brightness of the cpEGFP reporter. A recent breakthrough, leveraging a brighter fluorescent protein (mNeonGreen or mNG, possessing nearly 3‐fold higher brightness than EGFP), has led to the creation of a new series of ultra‐sensitive mNG‐based calcium indicators, designated as NEMO (Figure 1C).^10^ NEMO indicators are generated through iterative optimization of the calcium‐sensing module, the reporter, and the linkers connecting them. Five variants of NEMO with distinct optical properties have been created, with NEMOb, NEMOc, NEMOf, NEMOm and NEMOs representing bright, high‐contrast, fast‐acting, medium‐response, and sensitive versions, respectively. Distinguished from the GCaMP series, NEMO exhibits a spectral profile that lies between GFP and YFP, forming a narrower spectrum. This unique attribute enables imaging using either GFP or YFP filters, as well as multiplexing with CFP. Further enhancing its utility, NEMO sensors exhibit enhanced resistance to photobleaching under more intense illumination, effectively broadening their scope of in vivo application. Of paramount significance, the NEMO variants exhibit superior dynamic ranges (>100) and SBR values, reaching an unprecedented level of up to 220.

As aforementioned, a larger DR or SBR facilitates better resolution Ca^2+^ signal amplitudes. Indeed, NEMO sensors could resolve Ca^2+^ oscillations (Figure 2A) and store operated Ca^2+^ entry (SOCE) responses (Figure 2B) in non‐excitable cells, as well as Ca^2+^ transients in neurons (Figure 2C), with much larger SBRs. During high‐frequency electric field stimulation, NEMOc displayed a response amplitude exceeding that of GCaMP6 by more than 30 times. This remarkable improvement in SBR holds particular significance in deciphering signals within neurons or muscle cells, as the existing fast‐acting GECIs tend to have smaller in‐cellulo DRs. Prominent examples like GCaMP6f and jGCaMP7f possess DRs below 14 when expressed in mammalian cells, while the latest ultra‐fast variant, jGCaMP8f, registers a mere DR of 3.7. In contrast, NEMOf, exhibiting comparable speed to GCaMP6f and jGCaMP7f, has a DR that surpasses these two fast GECIs by 17 times. The substantial improvement in DR positions NEMOf as an ideal tool for effectively decoding both frequency and amplitude information within neural circuits or other excitable cells. Furthermore, NEMOf provides an excellent template for further engineering aimed at enhancing its kinetics to match or even surpass jGCaMP8f while still maintaining a reasonable DR.

**FIGURE 2:**
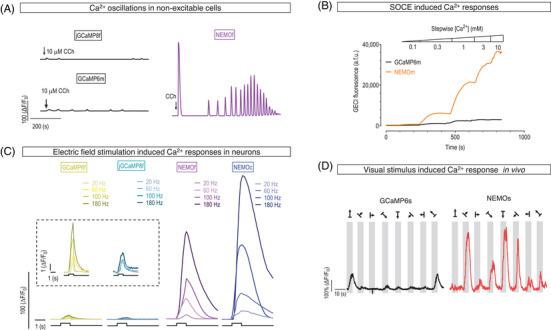
Typical traces showing performance NEMO indicators in resolving various types of Ca^2+^ signals. GCaMP6m/6s/6f, or jGCaMP8f were shown as controls (black, yellow or cyan traces，). (A) Ca^2+^ oscillations in HEK293 cells induced by carbachol (CCh). (B) Store operated Ca^2+^ entry (SOCE) responses of store‐emptied HEK293 cells bathed in solutions containing stepwise external Ca^2+^. (C) Electric field stimulation‐induced NEMO responses in rat hippocampal neurons. (D) In vivo Ca^2+^ responses of mice visual cortical neurons.

The extra‐large DR of NEMO indicators allows real‐time quantification of subtle Ca^2+^ transients with enhanced sensitivity. These NEMO sensors have undergone rigorous validation in diverse settings, encompassing non‐excitable cell lines, dissociated cultured rat neurons, acute mouse brain slices, in vivo two‐photon laser imaging (Figure 2D), fibre recording of mouse brains, and plant cells. Under each scenario, NEMO variants have shown superior performance over the GCaMP6 series. Furthermore, compared to the GCaMP series, NEMO indicators remain relatively stable within the pH range of 6–9. This feature imparts greater resilience to physiological pH fluctuations and renders them suitable for detecting Ca^2+^ signals within cancer cells under hypoxia conditions. By optimizing the Ca^2+^‐binding affinities and incorporating appropriate targeting sequences, modified NEMO indicators can be tailored to discern Ca^2+^ signals within acidic organelles like the endosomal compartments, as well as synaptic vesicles, secretory vesicles, and the trans‐Golgi apparatus. Looking ahead, subcellular NEMO indicators may also hold the promise of effectively resolving localized Ca^2+^ signals at membrane contact sites formed between the endoplasmic reticulum and mitochondria, or the plasma membrane.

In summary, in contrast to most existing GECIs, which often trade their DR for heightened sensitivity and faster kinetics, NEMO variants manage to maintain rapid responsiveness while preserving superior dynamic ranges for accurate Ca^2+^ signal reporting. Moreover, they exhibit substantially improved photostability, enabling them to withstand considerably stronger illumination, along with enhanced resistance to pH fluctuations. Collectively, these desirable characteristics position NEMO sensors as the preferred choice for monitoring Ca^2+^ dynamics within mammalian cells, tissues, living animals, as well as in plants.

## CONFLICT OF INTEREST STATEMENT

The authors declare no conflicts of interest.
